# The effect of strategy game types on inhibition

**DOI:** 10.1007/s00426-021-01632-0

**Published:** 2022-01-12

**Authors:** Aaron Yew Cheong Leong, Min Hooi Yong, Mei-Hua Lin

**Affiliations:** 1 Department of Psychology, Sunway University, 5 Jalan Universiti, 47500 Petaling Jaya, Selangor, Malaysia; 2 Department of Psychology, University of Bradford, Richmond Road, Bradford, BD7 1DP UK

## Abstract

Past studies have shown evidence of transfer of learning in action video games, less so in other types, e.g. strategy games. Further, the transfer of learning from games to inhibitory control has yet to be examined from the perspectives of time constraint and logic contradiction. We examined the effect of strategy games (puzzle, turn-based strategy ‘TBS’, and real-time strategy ‘RTS’) on inhibition (response inhibition and distractor inhibition) and cerebral hemispheric activation over 4 weeks. We predicted that compared to RTS, puzzle and TBS games would (1) improve response and distractor inhibition, and (2) increase cerebral hemispheric activation demonstrating increased inhibitory control. A total of 67 non-habitual video game players (*M*_age_ = 21.63 years old, SD = 2.12) played one of three games: puzzle (*n* = 19), TBS (*n* = 24) or RTS (*n* = 24) for 4 weeks on their smartphones. Participants completed three inhibition tasks, working memory (WM), and had their tympanic membrane temperature (TMT) taken from each ear before and after playing the games. Results showed that only the puzzle game group showed an improved response inhibition while controlling for WM. There were no significant changes in the distractor inhibition tasks. We also found that there was an increase in left TMT while playing RTS, suggesting the presence of increased impulsivity in RTS. Our findings suggest that puzzle games involving logical contradiction could improve response inhibition, showing potential as a tool for inhibition training.

## Introduction

In the past 30 years, video games have grown to be a popular form of leisure activity and e-sports. There is also a growing interest in how action video games such as *first-person shooter* (FPS) and *real-time strategy* (RTS) are associated with greater visual perception (Green & Bavelier, 2003; Oei & Patterson, 2015), selective attention (Bavelier et al., 2012; Qiu et al., 2018), and task switching (Basak et al., 2008; Dale & Green, 2017a; Glass et al., 2013). Evidence suggests that skills that were learned or trained further in video games could be observed outside of the video game context, for instance in surgery (Ou et al., 2013), piloting (McKinley et al., 2011), and military (Blacker et al., 2019).

The transfer of learning from games to inhibition tasks is less clear. Inhibitory control refers to the deliberate and controlled suppression of automatic, dominant, or initiated motor responses according to one’s goal (Friedman & Miyake, 2017). Greater inhibitory control would mean better regulation of thoughts, emotion, behaviour, motivation, and impulse (Hofmann et al., 2012). Games that improve inhibitory control could be used as a training tool to reduce impulsivity in monetary decisions (Oldrati et al., 2016; Stevens et al., 2015) as well as drinking and eating behaviour (Bartholdy et al., 2016; Hofmann et al., 2012).

The transfer of learning and skill improvement from a training task to an untrained task could occur when the tasks use similar skills or processing patterns (Taatgen, 2013). Playing strategy games involves planning, which shares similar processing patterns with two inhibitory functions: response inhibition and distractor inhibition. Response inhibition is the ability to monitor conflict by postponing, withholding, and cancelling preplanned actions based on the goal while distractor inhibition is the ability to resist the interference of stimuli unrelated to the goal (Friedman & Miyake, 2004). Past studies have shown that planning time and performance are positively correlated with response inhibition (Arfé et al., 2020; Asato et al., 2006; Zook et al., 2006) and distractor inhibition (Enticott et al., 2006) because the act of planning involves inhibiting impulsive decisions and irrelevant stimuli (Unterrainer & Owen, 2006). While playing action video games is known to improve visual perception, selective attention, and sensorimotor control, the non-action-strategy games such as *puzzle* games and *turn-based strategy* (TBS) games could potentially improve inhibition. We propose two possibilities to explain the transfer of inhibition; time constraint and logic contradiction.

### Time constraint

When playing a game, the available time becomes a variable in determining how players play the game. RTS games are fast-paced, requiring players to respond quickly and adaptively in real time. In contrast, puzzle games and TBS games generally do not have time constraints, allowing players to take time in planning their moves by simulating, evaluating, and revising the possible options to identify optimal solutions among many possible suboptimal or incorrect moves. This suggests that when given time, inhibition would be involved in inhibiting the selection of incorrect moves during planning (Arfé et al., 2020; Asato et al., 2006; Zook et al., 2006). Consistent with this, puzzle gameplay has been associated with slower but cautious perception task performance (Nelson & Strachan, 2009) and logical reasoning (Thompson et al., 2012). Similarly, chess players demonstrated higher accuracy and longer planning time before executing a move on a planning task than non-chess players (Unterrainer & Owen, 2006). This suggests that the absence of time constraint encourages deliberate planning, which shares similar processing with inhibitory control, thereby training inhibition control while reducing impulsivity.

Unlike puzzle games, RTS games have been shown to not affect response inhibition or distractor inhibition (Bailey et al., 2010; Basak et al., 2008; Oei & Patterson, 2014). As time constraint limits planning (Gray et al., 2006; Liberman & Trope, 1998), the nature of RTS games which requires quick hand–eye coordination prohibits deliberate and cautious planning. However, not much is known about the effect of TBS games on inhibition. TBS games do not have time constraints unlike RTS and, similar to puzzle games, enables players to plan before making a move and consider alternative or more efficient solutions (Dale & Green, 2017b; Shafer, 2013). If action-strategy RTS games do not improve inhibition because of their real-time gameplay, TBS games are likely to improve inhibition.

### Logical contradiction

In addition to only considering puzzle games that have no time constraints, we are interested in examining the subset of puzzle games that (1) have a limited number of optimal solutions and (2) are designed with logical contradictions that lure players into making incorrect assumptions (Brown 2018; Menzel, 2016; Poole, 2004; Schell, 2015). Although these qualities are not present in all games that would generally be labelled as “puzzle” games in the field, these mechanics are commonly utilised across the genre. The logical contradiction in puzzle games is similar to the goal–subgoal conflicts in the planning tasks Tower of London and Tower of Hanoi. Past evidence has shown that the planning process includes making seemingly counterintuitive moves (i.e. making a move that conflicts with the goal) to achieve the goal, which requires inhibiting automatic but incorrect responses (Asato et al., 2006; Kaller et al., 2011; Welsh et al., 1999; Zook et al., 2006). In the case of the puzzle game Flow (Big Duck Games, 2012) used in the present study, the game misdirects players by presenting players with the same coloured dots in close proximity and that connecting these dots via a direct path would block a path for a different pair of dots. Solving this puzzle would require the players to inhibit their automatic response (i.e. connect the coloured pairs via a direct path) and make counterintuitive moves (i.e. connect the dots via an indirect path) to resolve the logical contradiction. Therefore, puzzle game players may be trained to inhibit the automatic response to these lures, and this training improves response inhibition.

Unlike puzzle games, RTS and TBS games do not have logical contradictions in the game design. This could be attributed to the multiple optimal solutions in RTS and TBS games. The stages in these games do not have a single optimal solution, which encourages players to adapt and make decisions from a large number of possibilities. For example, in the TBS game Warlords of Aternum (InnoGames GmbH, 2015), players may choose five of the ten units in any combination for each stage, choose different placement and movement for each unit, and choose different possible combinations of upgrades for each unit. Past research has found that the number of optimal solutions in a task negatively correlates with planning time (Unterrainer et al., 2006), which positively correlates with inhibition (Arfé et al., 2020; Asato et al., 2006). This suggests that the greater number of possible solutions in RTS and TBS games could lead to a reduction in planning and consequently less training of response inhibition.

### Working memory

Other than time constraint and logic contradiction, working memory (WM) is a possible factor in inhibition performance. Past studies have shown that having better WM is associated with better performance in RTS games (Basak et al., 2008; Glass et al., 2013) and puzzle games (Thompson et al., 2012) because players need to maintain and update information while planning simultaneously in the games. Further, higher WM is related to greater inhibitory control because WM maintains the task instruction during inhibition tasks, guiding the selection of appropriate actions and inhibiting incorrect actions (Conway et al., 2001; Kane & Engle, 2003). These studies suggest playing strategy video games may improve inhibitory control because of the involvement of WM in the planning of subsequent actions.

#### Hemispheric activation

To our knowledge, the effect of non-action video games on cerebral activation has not been examined. Games that improve inhibition could also affect hemispheric activation associated with inhibition. This could be inferred by measuring tympanic membrane temperature (TMT), where the difference between right TMT and left TMT (ΔTMT) reflects the difference in hemispheric activation. Individuals with greater inhibition were found to have a more positive ΔTMT (right TMT minus left TMT), indicating a greater right hemispheric activation than the left hemispheric activation (Helton, 2010). In contrast, individuals with weaker inhibition or greater impulsivity would have a more negative ΔTMT, indicating a greater left hemispheric activation (Balconi et al., 2015; Helton & Maginnity, 2012). Therefore, strategy games that improve inhibition could also increase ΔTMT. It is of interest to investigate whether non-action games could also affect hemispheric activation (as measured using ΔTMT) as one study has shown an improvement in inhibition over 4 weeks (Oei & Patterson, 2014).

#### The present study

Our overarching aim was to examine the effects of types of strategy games on response and distractor inhibition, as well as hemispheric activation. To our knowledge, the comparison of time constraint and logic contradiction found in games has yet to be examined. Further, having higher WM is associated with improved inhibition, suggesting that WM may be a confound on inhibition performance. Based on the literature above, we hypothesised that (H1) there would be a greater improvement in response and distractor inhibition in the puzzle group and TBS group compared to the RTS group, and (H2) there would be an increase in ΔTMT in the puzzle group and TBS group compared to the RTS group.

## Methods

### Participants

We recruited a total of 102 participants: randomly assigned into RTS (*n* = 36), TBS (*n* = 36), and puzzle (*n* = 33) conditions. Inclusion criteria included (1) were non-habitual video game player (NVGP) with playing less than 1 h of video games per week in the past 12 months or casual player with playing less than 1 h of RTS, TBS, and puzzle games and less than 3 h of any other games (e.g. simulation games) per week in the past 12 months, (2) aged between 19 and 40 years old, (3) owned a smartphone, (4) right-handed or ambidextrous, (5) had normal or corrected to normal vision, and (6) did not have a confirmed diagnosis of psychiatric condition, neurological condition, or substance abuse. After removing some attrition (*n* = 35), our final sample was 67 participants (RTS = 19, TBS = 24, puzzle = 24). Our participants were mainly female (89.5%) with a mean age of 21.63 years (*SD* = 2.12) (see Table 1 for details). We obtained ethical approval from university research ethics committee (approval code: PGSUREC 2019/019) and participants provided consent prior to participating. Participants were provided with travel reimbursement on both occasions and a small token of appreciation.

**Table 1:** Descriptive and inferential statistics for participants at baseline (*n* = 67)

	RTS (*n* = 19)		TBS (*n* = 24)		Puzzle (*n* = 24)		ANOVA		
	*M*	*SD*	*M*	*SD*	*M*	*SD*	*F*	*p*	Partial η^2^
Age	21.26	2.68	21.73	1.58	21.43	1.78	0.44	0.645	0.01
Sex	17F/2M		20F/4M		23F/1M				
Handedness	18R/1A		22R/2A		22R/2A				
Phone screen size (diagonal measurement in cm)	14.07	1.41	13.81	1.85	13.51	1.69	0.41	0.668	0.01
Time spent playing games in the past 12 months (in hours)	2.95	4.38	2.94	4.09	3.10	4.93	0.01	0.990	0.00
Type of video game players	12 NVGP/7 casual		14 NVGP/10 casual		18 NVGP/6 casual				

*F female, *M* male, *R* right-handed, *A* ambidextrous

### Materials

#### Games’ selection

For game selection, we recruited 21 young adults (12 females; *M*_age_ = 24.90, SD = 2.00) and they were randomly assigned to 1 game category (e.g. RTS) to which they played and reviewed 4 RTS games for an hour per game in a week. The four games were selected based on the following criteria: (a) the game description fits the game type (e.g. in the case of puzzle games, the game needed to be labelled as a “puzzle game” and also include no time constraints, a limited subset of solutions, and the regular presence of logical contradictions); (b) the game had at least 4.3 stars ratings with 40,000 reviews on Google Play Store as of 21st February 2019; (c) the game consisted of single-player option and progressed in stages; and (d) the game was available for free on android phones and iOS phones. After playing the games, we selected those with the highest mean ratings on perceived strategic thinking and game experience for each game from our testers (i.e. RTS: Galaxy Reavers (Good Games LLC, 2016), TBS: Warlords of Aternum (InnoGames GmbH, 2015), puzzle: Flow (Big Duck Games, 2012); see Table 2 for full details). The three chosen games did not significantly differ in terms of game experience, *F*(2, 18) = 1.51, *p* = 0.249, and strategy, *F*(2, 18) = 2.70, *p* = 0.093.

**Table 2:** Perceived strategic thinking and game experience for each game

Type of game and ANOVA	Strategic thinking		Game experience	
	*M*	*SD*	*M*	*SD*
RTS (*n* = 8)				
Galaxy Reavers	6.75	2.12	6.93	2.35
Age of Ottoman	6.63	2.45	5.35	1.26
The Horus Heresy: Drop Assault	6.38	2.77	6.17	2.35
Art of War 3	4.25	2.91	5.40	1.94
TBS (*n* = 7)				
Warlords of Aternum	8.57	0.79	8.24	1.45
Uniwar	8.23	0.95	7.71	1.95
Tactical Monsters Rumble Arena	7.43	1.62	7.19	2.1
King’s Bounty Legions	6.43	1.81	4.74	1.13
Puzzle (*n* = 6)				
Flow	8.33	1.63	8.30	0.51
Cut the Rope Time Travel	7.67	0.82	7.96	1.25
Cut the Rope	7.50	0.84	7.04	1.37
Cut the Rope 2	7.33	2.42	7.13	2.84

* Those in bold were selected for the study

#### Inhibition tasks

There were three tasks: the stop-signal task (response inhibition), and the Stroop task and the Multi-Source Interference Task (MSIT), both to measure distractor inhibition. All tasks were completed on a Windows computer (with a 60 Hz frame rate) in the research laboratory.

*Stop-Signal Task.* We used the stop-signal task on the STOP-IT software (Verbruggen et al., 2008). On each trial, one of two shapes (square or circle) was displayed in the middle of the screen with a black background for 1250 ms (ms) or until participants have responded. Participants were informed to press “z” for square and “/” for circle. The task consisted of 32 practice trials and 3 blocks of 64 trials each with an equal frequency for both shapes. There were 16 stop-signal trials in each block, in which participants were to withhold their response when the stimulus was followed by an auditory signal (a 75 ms beep). The auditory signal was set at 250 ms after the stimulus. This delay, called the stop-signal delay (SSD), increased by 50 ms following successful trials and decreased by 50 ms following unsuccessful trials. This SSD setting maintained the probability of responding on a stop-signal trial (*p*[respond|signal]) for each participant at 50% to produce the most reliable inhibition estimates. A fixation cross was displayed for 250 ms between each trial. Stop-signal task performance was measured using the stop-signal reaction time (SSRT). SSRT was calculated from correct responses using the following formula: SSRT = mean of go-RT (trials without stop-signal) minus the mean of SSD. A lower SSRT indicates greater stop-signal task performance, which reflects greater response inhibition.

#### Stroop task

The colour Stroop task was programmed on Psychopy version 1.90.1 (Peirce et al., 2019). On each trial, a word (either “red”, “blue”, “green”, or “yellow”) was displayed in the middle of the screen for 5000 ms or until participants have responded. Participants were required to press the appropriate key ‘q’ for red, ‘p’ for blue, ‘z’ for green and ‘m’ for yellow on the keyboard. A paper cover was used to cover the entire keyboard except for the response keys. There were a total of 24 practice trials and 4 blocks of 96 trials. Within each block, the font colour was incongruent for half of the trials. Stroop performance was measured using the average RT (in ms) of correct responses on the incongruent trials, where a lower RT indicates greater Stroop performance, which reflects greater distractor inhibition.

#### Multi-source interference task

Participants completed a number-variant version of the MSIT (Bush et al., 2003). For each trial, one set of three numbers (0, 1, 2, 3) was displayed in the middle of the screen, and participants were informed to respond to the unique number using the corresponding number on the keyboard. Participants were informed that one number will be unique compared to the other two and therefore to press that matching numeral key while ignoring the position and font size of the numbers which may distract the participants from entering the correct response. Participants completed 20 practice trials before completing 100 trials for the main task. Half of the trials were congruent, in that the correct response was in the same position while the other half was incongruent. Each trial lasted for 3000 ms. MSIT performance was measured using the average RT (in ms) of correct responses in incongruent trials, where lower RT indicates greater MSIT performance, which reflects greater distractor inhibition.

#### Tympanic membrane temperature

An infrared ear thermometer (model: Beurer FT 58, Germany) was used to measure the TMT. Three TMT readings were recorded on each ear immediately before and after each of the inhibition tasks (a total of 18 TMT readings for each ear in pretest and posttest, respectively). ΔTMT was calculated as the average difference in TMT between right ear and left ear (ΔTMT = Σ(right TMT—left ear TMT)/18). A more positive value of ΔTMT indicates higher right TMT relative to left TMT, which reflects greater cerebral activation in the right hemisphere compared to the left hemisphere.

*Working Memory.* WM was measured using the operation span (OSPAN) task on the CogLab software (Francis et al., 2008). On each trial, participants were shown a mathematical equation (e.g. “is (5/1) + 4 = 9?”) and indicated whether the equation is correct or incorrect by clicking the options provided on the screen. A word was then shown on the screen (e.g. “bench”). There was a total of two practice trials and 60 trials. The trials were grouped into 15 sets, whereby each set varied between two to six trials. At the end of every set, participants were asked to recall the sequence of the words’ appearance by clicking on the options provided on-screen. WM was measured using the sum of words recalled correctly across the 15 sets. The sum ranged from 0 to 60, where a higher sum reflects greater WM.

#### Additional measures

Participants completed questions on game satisfaction, perceived similarity, and perceived strategic thinking. We measured game satisfaction using six subscales of the Game User Experience Satisfaction Scale (GUESS) on a 7-point Likert scale with one ‘strongly disagree’ to seven ‘strongly agree’ (Phan et al., 2016), which were usability, play engrossment, enjoyment, audio aesthetics, personal gratification, and visual aesthetics. We created a 4-item scale to measure the perceived similarity between the type of game played and each inhibition task. An example is “I found the task to be as challenging as the game”. We also measured perceived strategic thinking for each game using a single-item question (i.e. “This game requires strategic thinking.”). For perceived similarity and strategic thinking scales, participants responded on a Likert scale ranging from one (*not true at all*) to seven (*very true*). Higher average scores on these measures indicate greater game satisfaction, perceived similarity, and perceived strategic thinking.

### Procedure

At pretest, participants completed the OSPAN task and followed by the three inhibition tasks. The order of the inhibition tasks was counterbalanced. We also recorded participants’ TMT measurements before and after each inhibition task. Participants were given a short break between each task. Participants were then informed to download one of the three games onto their smartphone and were informed to play 1 h each day, 5 days a week, for 4 weeks. They were also informed not to play the game on other devices, e.g. tablets or computers and to complete their game progress log.

At the end of the 4 weeks, participants again completed the three inhibition tasks and had their TMT measured. Participants also completed the GUESS and submitted the game progress log that tracked the time spent playing.

### Data analysis

We first screened participants’ game progress logs to ensure that participants have completed 20 h of training with at least 10 sessions as well as each training session was at least 30 min and did not exceed 2 h to ensure that participants followed our training guidelines. Participants’ total time spent playing the assigned game and time spent playing in each sitting were not significantly different across groups (*p*s > 0.05). Next, we conducted trial-level data cleaning to ensure all responses were above the threshold of anticipatory responses (i.e. 250 ms) and below the maximum response duration on each task (i.e. 3000 ms for the MSIT, and 5000 ms for the Stroop task). We then analysed the data for unreliable data and outliers at the participant level using 5% trimmed means and boxplots. We also used Shapiro–Wilk tests and scatterplots for normality, homogeneity of regression slopes, and homoscedasticity; Levene’s tests were conducted to check for the homogeneity of error variances. We also examined whether WM was a covariate in the effect of type of game on inhibition by conducting bivariate correlations between WM and all measures of inhibition. We then used analysis of covariance (ANCOVA) to test our hypotheses. Simple main effect analysis was conducted to follow-up on significant interaction effect. Speed-accuracy trade-off was also examined for significant interaction effect.

## Results

### Working memory and inhibition

From our correlation analysis, our results showed that WM was negatively correlated with stop-signal task performance and Stroop task performance, but not MSIT task performance or ΔTMT (see Table 3). The inhibition tasks were positively correlated with each other (all *p*s < 0.01). Therefore, we included WM as a covariate in examining response inhibition and distractor inhibition.

**Table 3:** Descriptive statistics and intercorrelations for inhibition measures and OSPAN performance at pretest

Variable	*M*	*SD*	1	2	3	4	5	6	7
1. OSPAN performance	48.61	9.88	–						
2. SSRT	282	32	− 0.36**	–					
3. Go-RT	580	120	− 0.27*	0.22	–				
4. MSIT congruent RT	650	133	− 0.03	0.30*	0.07	–			
5. MSIT incongruent RT	966	156	− 0.20	0.25	0.08	0.81**	–		
6. Stroop congruent RT	677	86	− 0.27*	0.36**	0.40**	0.54**	0.63**	–	
7. Stroop incongruent RT	762	102	− 0.28*	0.28*	0.37**	0.42**	0.56**	0.42**	–
8. ΔTMT	− 0.07	0.14	0.00	0.00	0.05	-0.02	0.13	0.10	0.01

*p < .05; **p < .01

### Response inhibition

#### Stop-signal task performance

**Fig. 1:**
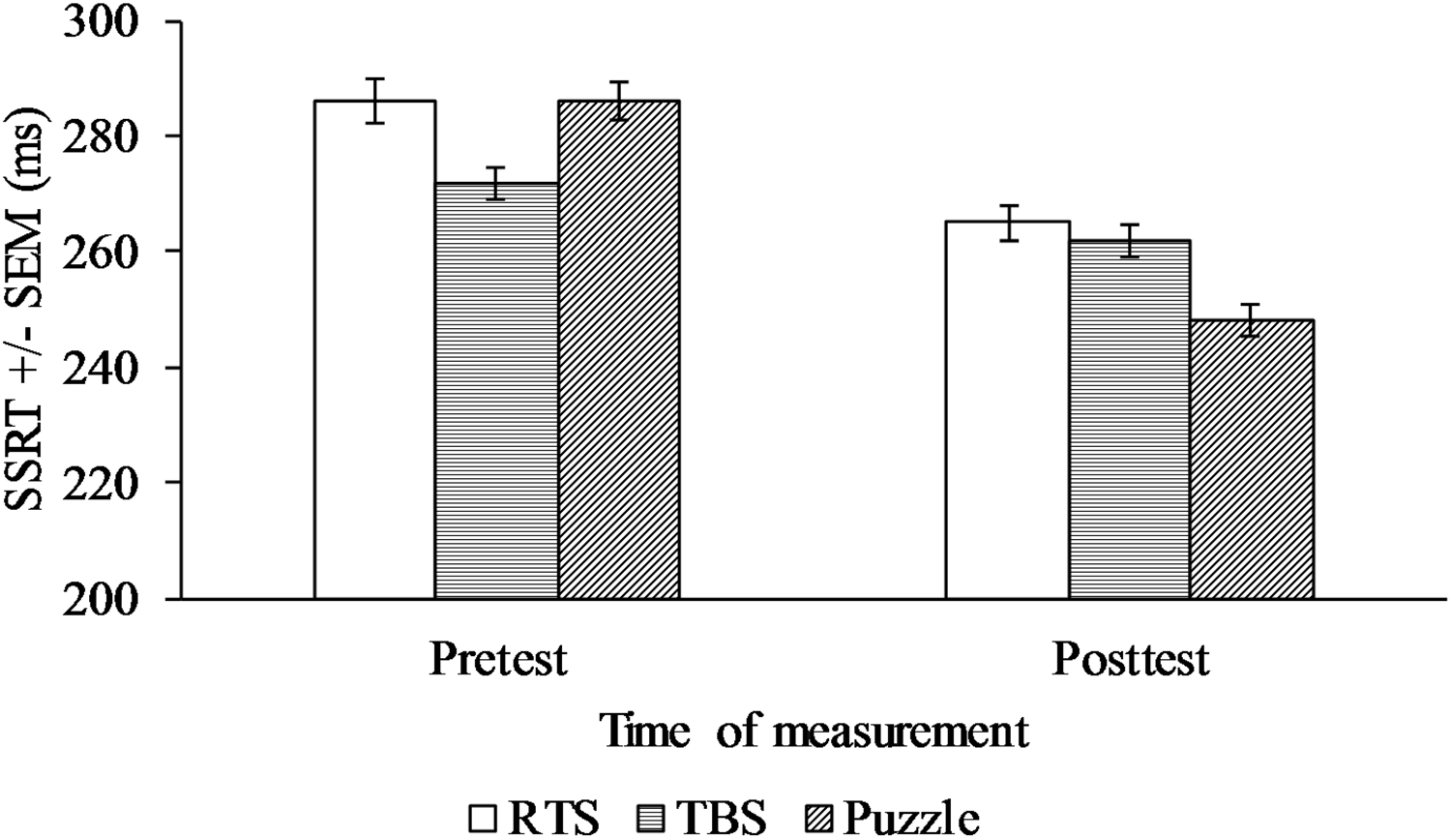
Mean RT for response inhibition task performance across groups at pretest and posttest

A 3 (Game Type: RTS, TBS, Puzzle) × 2 (Time: Pretest, Posttest) mixed ANCOVA was conducted with WM as a covariate. The effect of WM on SSRT was significant, *F*(1, 54) = 5.34, *p* = 0.025, partial *η*^2^ = 0.09 indicating that WM exerted its influence on SSRT performance. The main effect of type of game was not significant, but SSRT significantly decreased from pretest (*M* = 281 ms, *SE* = 3.94) to posttest (*M* = 258 ms, *SE* = 3.29). The interaction effect was also significant, *F*(2, 54) = 3.93, *p* = 0.026, partial *η*^2^ = 0.13 (see Fig. 1).

We followed up with one-way repeated measures ANCOVAs. SSRT significantly improved in the puzzle group, *F*(1, 19) = 5.73, *p* = 0.027, partial η^2^ = 0.23, but not in the RTS group and the TBS group (*p*s > 0.05).

To examine whether the improvement in stop-signal task performance in the puzzle group could be due to a speed-accuracy trade-off, we conducted 3 × 2 mixed ANCOVAs on go-trials RT and accuracy (i.e. percentage miss in go-trials) while controlling for WM. A speed-accuracy trade-off would be indicated by a lower go-trials RT and a lower accuracy only in the puzzle group. We only found a significant effect of WM on go-RT, *F*(1, 54) = 4.39, *p* = 0.041, partial *η*^2^ = 0.08. The interaction effects and the main effects were not significant (all *p*s > 0.05). Overall, the results indicated no evidence for speed-accuracy trade-off on the stop-signal task performance (Table 4).

**Table 4:** Simple main effects for stop-signal task performance with and without WM as a covariate

Group	Effect	*F*	*df*1	*df*2	*p*	Partial η^2^
No covariate						
RTS	Time	9.25	1	15	0.008	0.38
TBS	Time	3.66	1	21	0.070	0.15
Puzzle	Time	16.30	1	20	0.001	0.45
With covariate						
RTS	Time	0.41	1	14	0.533	0.03
	WM	6.25	1	14	0.025	0.31
TBS	Time	0.01	1	19	0.933	0.00
	WM	1.04	1	19	0.321	0.05
Puzzle	Time	5.73	1	19	0.027	0.23
	WM	0.33	1	19	0.576	0.02

The results for the puzzle group and TBS group do not differ with and without WM as a covariate

To examine whether the results would differ when WM was not included as a covariate, we conducted a 3 (Game Type) × 2 (Time) mixed ANOVA on SSRT where WM was not included as a covariate. The interaction was significant when WM was not a covariate, *F*(1, 56) = 4.59, *p* = 0.014, partial *η*^2^ = 0.14, similar to when WM was a covariate. However, the simple main effect analysis was different without WM as a covariate. Here, we found that participants in the RTS group showed a significant improvement from pretest (*M* = 287 ms, *SE* = 8 ms) to posttest (*M* = 265 ms, *SE* = 6 ms), *F*(1, 15) = 9.25, *p* = 0.008, partial η^2^ = 0.38. In contrast, when WM was included as a covariate, the RTS group showed a non-significant improvement (see Table 5 for a summary of the simple main effect analysis with and without WM as a covariate). This difference of result for the RTS group could be due to the involvement of WM in playing RTS games, suggesting that WM is related to the RTS game and could act as a covariate in the present study.

**Table 5:** Descriptive and inferential statistics on perceived similarity, strategic thinking, and game satisfaction (*n* = 67)

Measure	RTS (*n* = 19)		TBS (*n* = 24)		Puzzle (*n* = 24)		ANOVA		
	*M*	*SD*	*M*	*SD*	*M*	*SD*	*F*	*p*	Partial η^2^
Perceived similarity									
Stop-signal task	2.95	1.37	3.06	0.99	2.98	1.44	0.04	0.964	0.00
MSIT	2.84	1.81	3.03	0.94	3.07	1.55	0.15	0.864	0.01
Stroop task	2.79	1.50	3.02	1.08	3.86	1.50	3.69	0.031	0.11
Perceived strategic thinking	5.53	1.81	6.08	0.78	5.96	1.72	0.80	0.454	0.02
Game satisfaction	4.26	0.97	5.16	0.74	5.16	0.83	7.93	0.001	0.20

### Distractor inhibition

#### Stroop task performance

**Fig. 2:**
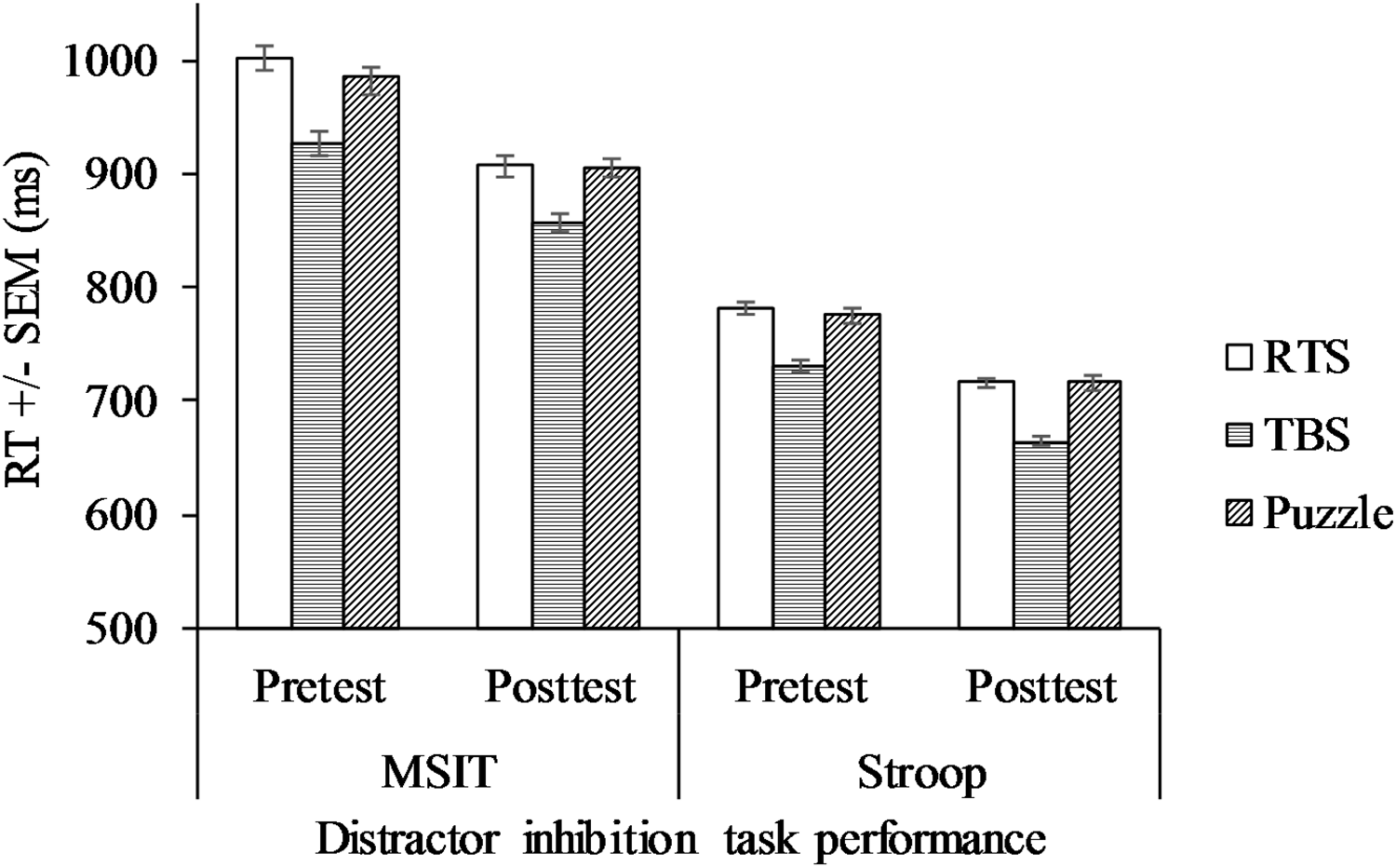
Mean RT for distractor inhibition task performance across groups at pretest and posttest

We used a similar 3 × 2 ANCOVA analysis for the Stroop task. Similar to SSRT, the effect of WM on Stroop incongruent RT was significant, *F*(1, 60) = 4.79, *p* = 0.033, partial *η*^2^ = 0.07. We did not find a significant interaction effect or main effects of time and game type (all *p*s > 0.071) (see Fig. 2).

#### MSIT performance

We used a 3 × 2 ANCOVA analysis for the MSIT task. Unlike the SSRT and Stroop, the effect of WM on MSIT incongruent RT was not significant, *F*(1, 60) = 2.03, *p* = 0.160. We did not find significant interaction effect or main effects on game type and time (all *p*s > 0.092) (see Fig. 2).

### Tympanic membrane temperature

**Fig. 3:**
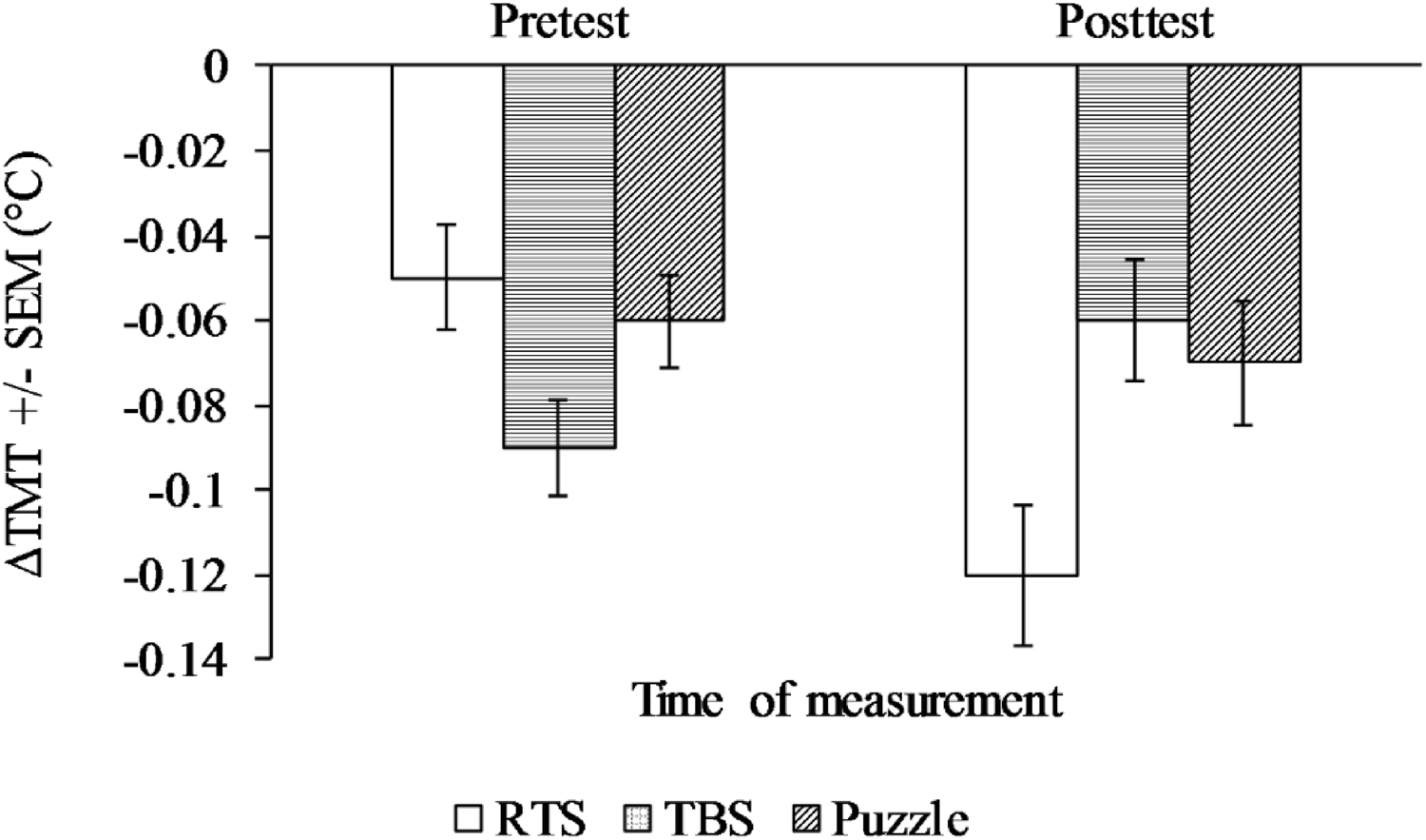
Mean ΔTMT across treatment groups at pretest and posttest

We conducted a 3 (Game Type) × 2 (Time) mixed ANOVA. Results showed that the interaction effect on ΔTMT was significant, *F*(2, 55) = 4.19, *p* = 0.020, partial *η*^2^ = 0.13. However, we did not find significant main effects of time, *F*(1, 55) = 0.74, *p* = 0.394, partial *η*^2^ = 0.01, and game type, *F*(2, 55) = 0.22, *p* = 0.805, partial *η*^2^ = 0.01 (see Fig. 3).

Simple main effect analysis was conducted using one-way repeated measures ANOVAs as follow-up to the interaction effect. ΔTMT significantly decreased in the RTS group, *F*(1, 15) = 11.25, *p* = 0.004, partial *η*^2^ = 0.43, but not in the puzzle group, *F*(1, 20) = 0.06, *p* = 0.815, partial *η*^2^ = 0.00, and the TBS group, *F*(1, 20) = 2.67, *p* = 0.118, partial *η*^2^ = 0.12.

We also analysed game satisfaction, perceived similarity, and perceived strategic thinking using one-way between-subjects ANOVA (see Table 4). For perceived strategic thinking, there was no significant different across three game types, *F*(2, 65) = 0.80, *p* = 0.454. There was significant difference for game satisfaction, *F*(2, 65) = 7.93, *p* = 0.001, partial *η*^2^ = 0.20, to which RTS game was rated significantly lower in-game satisfaction compared to the TBS game (*p* = 0.003) and the puzzle game (*p* = 0.002). As for perceived similarity, there was no significant difference for stop-signal and MSIT, both *p*s > 0.864. However, the perceived similarity for the Stroop task was significantly different across groups, *F*(2, 62) = 3.69, *p* = 0.031, partial *η*^2^ = 0.11, to which puzzle game was rated significantly more similar to the Stroop task than the RTS game (*p* = 0.031).

## Discussion

We hypothesised that the absence of time constraints (i.e. more planning time at leisure) in strategy games would be beneficial in training response inhibition, and our results showed that this was the case only for the puzzle game after controlling for WM. Our findings are concordant with Oei and Patterson’s (2014) findings, in that we found puzzle games improved response inhibition (stop-signal task performance) but not for RTS and TBS games. We are also certain that our results were not attributable to a speed-accuracy trade-off, as evidenced by our non-significant results. The results are also not attributable to individual variation in terms of perceived strategic thinking, perceived similarity between game and stop-signal task, or game satisfaction.

We did not find evidence of response inhibition improvement in the TBS game, another game with no time constraint similar to the puzzle game. One possibility is the presence of logical contradiction, commonly found in puzzle games but not in TBS games. Games with logical contradiction would train inhibition further, as ‘mistakes’ would lead to incorrect moves and encourage players to inhibit their impulsiveness. This is because puzzle game often leads players into making incorrect assumptions, thus they have learned to inhibit their assumptions to make counterintuitive moves.

Unlike response inhibition, we did not find any significant difference in the distractor inhibition tasks. These strategy games may require players to utilise all information and options provided on the user interface and the playing field for optimal moves or completion of the goal, thus requiring them to remain attentive to all visual cues instead of inhibiting non-relevant stimuli. To perform optimally in the RTS game accurately, players would need to accurately keep track of enemy units and abilities while managing their own units and abilities in real-time. This improvement in attention to peripheral cues could be transferred to visual attention tasks such as multiple object tracking but not distractor inhibition (Boot et al., 2013; Cain et al., 2012). Likewise, puzzle and TBS games similarly require players to attend to all available information to complete the in-game objectives. Players were likely to have done this to progress in the game, therefore, the training period would not have trained distractor inhibition.

In addition, we found that our results for response inhibition differed when WM as included as a covariate, in that we found no response inhibition improvement in the RTS game only when WM was covaried out. These results indicate that when RTS players were monitoring conflict while planning a goal simultaneously, they were dependent on WM for response inhibition in the RTS game. The RTS game requires players to constantly monitor their own and enemies’ moves in real-time to progress thus a higher WM would lead to better overall game performance. Unlike the RTS game, TBS and puzzle games do not face similar time constraints and would be able to obtain optimal game performance even with leisurely planning time, thus requiring less WM. This is consistent with past research that showed RTS gameplay to be strongly correlated with WM (Basak et al., 2008; Boot et al., 2008; Cardoso-Leite et al., 2016; Colzato et al., 2013; Nouchi et al., 2013; Sala et al., 2018; Thompson et al., 2012). Therefore, we have decided to control for WM to show the variance in inhibition that is uniquely explained by game type.

Past studies have shown that WM is associated with inhibition tasks (Conway et al., 2001; Kane & Engle, 2003; Welsh et al., 1999), and our results were similar to past studies in that, higher WM leads to greater response inhibition and distractor inhibition. However, when WM was included as a covariate in our analyses, we found that WM was significant in response inhibition (SSRT) and inconsistent in the distractor inhibition tasks—significant in Stroop but not significant in MSIT. Although both Stroop and MSIT tasks require participants to inhibit their responses, participants could have simply resisted the interference by not coding the information to memory, therefore, using less WM. Reading coloured words and numbers is relatively easy and with more incongruent trials, participants may have learned to inhibit their responses better (Meier & Kane, 2013; Ortells et al., 2017).

As for hemispheric activation, our results showed a decrease in ΔTMT in the RTS group, but no changes in the TBS group or puzzle group. This decrease meant that the left TMT (indicative of left hemispheric activation) increased relative to the right TMT (indicative of right hemispheric activation), suggesting a decrease in inhibition. Why is this so? One reason could be an increase in impulsivity and risk-taking. Individuals with greater impulsivity and risk-taking tendencies have been shown to have more negative values of ΔTMT (i.e. higher left hemispheric activation) (Balconi et al., 2015; Helton & Maginnity, 2012). In RTS games, swift use of various abilities would be more rewarding than a cautious approach because players could defeat an enemy unit before it becomes a threat and reactivate their abilities quicker after the ability cooldown. Therefore, RTS players are likely to be more impulsive for they require to make fast decisions for immediate payoffs.

Contrary to our prediction, the puzzle game and TBS game did not increase right hemispheric activation relative to left hemispheric activation. Participants in the puzzle and TBS groups may have shown striatal activity in the midbrain. Striatal activity is negatively correlated with impulsive decision making (Pan et al., 2021). Puzzle and TBS games encourage players to take a cautious approach by considering the layout of the puzzle or positioning of friendly and enemy units, rewarding players for completing the stages efficiently with a minimal number of moves. Therefore, puzzle and TBS players would be less impulsive and may show greater striatal activity.

There are two possible limitations to the present study. First, we did not control for two game characteristics that could have led to greater training gains in the puzzle group—in-game feedback and difficulty level adjustment (Dörrenbächer et al., 2014; Howard-Jones et al., 2016). The puzzle game provides clear feedback in the form of high scores and allows players to choose their puzzle difficulty. These characteristics allow players to choose puzzle stages that balance between players’ skills and puzzle difficulty. These characteristics could help players to be more engaged and focused in playing the puzzle game continuously, gradually improving in the puzzle game and response inhibition. This is not available in TBS for players start from the beginning, therefore TBS games are dependent on skills rather than selecting what might work best. Further, manipulation of this game feature within a single type of puzzle game could increase experimental control, minimising the differences between types of games.

Second, we did not control for the level of complexity across the three games. Specifically, RTS games may be too technical and competitive for non-habitual players. We found that non-habitual players (those who remained in the study and those who have withdrawn) rated the RTS game lower in-game satisfaction compared to the TBS and puzzle games. Participants also commented that they did not enjoy playing or had difficulty understanding to play the assigned game. Non-habitual players may have had a more difficult time in understanding the technical gameplay of the Galaxy Reavers RTS game (Good Games LLC, 2016; e.g. passive and active abilities, movement speed, cooldown, damage, barrier, positioning) than that of the Warlords of Aternum TBS game (InnoGames GmbH, 2015) which uses a rock-paper-scissors mechanic (e.g. guardians beat pikes; pikes beat mounted; mounted units beat guardians). Consistent with this, non-habitual players have been found to prefer simpler games and spend less time playing competitive and complex action games such as RTS games than habitual players (Limelight Networks, 2019, 2020, 2021). The technical gameplay, coupled with the real-time element, of RTS games may be a steeper learning curve for non-habitual players. Therefore, RTS game training could lead to lower compliance and higher attrition compared to TBS and puzzle game training.

### Application

Our findings suggest that puzzle games could be used to train response inhibition. Clinicians could explore the use of puzzle games in training individuals with impaired response inhibition, such as older adults (Basak et al., 2008) and individuals with attention-deficit-disorder ADHD (Johnstone et al., 2010). Puzzle games are typically designed as a form of entertainment, which could increase compliance among such individuals. Other than this, our findings could be applicable to the military with regards to shooting. One of the steps involved in shooting a firearm is making shoot/do not shoot decisions, which is positively correlated with response inhibition (Hamilton et al., 2019). Puzzle games could be a suitable alternative over action games, e.g. RTS games, which have been proposed to improve shoot/do not shoot speed and accuracy (Blacker et al., 2019) because action games may be associated with impulsive and risky decision making.

## Conclusion

In conclusion, when controlling for WM, we found that only puzzle games improve response inhibition, suggesting that planning in puzzle games could train players in inhibiting their preplanned responses. The effect was not significant for distractor inhibition, suggesting that changes in one measure of inhibition are not necessarily accompanied by a change in another. RTS game increased left hemispheric activation relative to right hemispheric activation, which is indicative of increased impulsivity and risk taking. Future research could further examine the planning process in these games and their potential in inhibition training.
